# Verifying monitor unit calculations for tangential whole‐breast fields in three‐dimensional planning

**DOI:** 10.1120/jacmp.v9i1.2713

**Published:** 2008-01-28

**Authors:** Ian Kay, Tyler Meyer

**Affiliations:** ^1^ Department of Medical Physics Tom Baker Cancer Centre; ^2^ Department of Oncology University of Calgary; ^3^ Department of Physics and Astronomy University of Calgary Calgary Alberta Canada

**Keywords:** Breast, tangent field, treatment planning, quality assurance, monitor‐unit calculation

## Abstract

Verification of the accuracy of monitor unit calculations is an essential component of quality assurance in radiation therapy. For tangential breast fields, monitor unit differences between primary calculations and second checks are usually larger than would be considered acceptable at other anatomic sites. Here, we present a simple model to reconcile the differences between sophisticated and simple algorithms, based on estimating the volume irradiated by the field, replacing the breast contour with a rectangular block having an equal volume, but using a new field width that provides almost equivalent scatter to the prescription point. This analysis can also assist the treatment planning physicist in selecting a tolerance window for verifying monitor unit calculations for tangential breast fields.

PACS numbers: 87.53.Kn, 87.53.Tf, 87.53.Xd

## I. INTRODUCTION

Quality assurance (QA) for external‐beam therapy requires that plans be validated,^(1)^ and an independent monitor unit calculation is an essential part of that QA process. Tangent fields used for breast treatment always present a challenge to the physicist performing plan checks, particularly the verification of monitor units. Breast tangent fields combine complex external contours and field borders outside the body, resulting in a significant amount of “missing tissue.” Monitor unit verification is usually performed with simple algorithms that assume full scatter conditions. The result is an overestimate of scatter dose to the calculation point in the second check and an underestimate of the monitor units required to deliver the desired dose.

Various algorithms—for example, Ayyangar et al.^(2)^—have been devised to estimate and remove the discrepancy. Kay and Dunscombe^(3)^ presented a simplified method for 2.5‐dimensional (2.5D) breast tangent fields. Their method estimates the cross‐sectional area of irradiated breast and determines the field dimensions that would irradiate a rectangle of equal area. Using the original and revised field widths, a correction factor is then calculated based on a ratio of phantom scatter factors $\left(S_{p}\right)$ and the tissue maximum ratios (TMRs). The accuracy of that correction factor is similar to that of the factor derived by Ayyangar et al.,^(2)^ and it is simpler to calculate. Prado et al.^(4)^ proposed a similar technique using a triangular approximation of the superior–inferior external breast contour as seen in a beam's eye‐view. Their method corrects monitor unit calculations based on full scatter conditions to within 2% of those calculated using a three‐dimensional (3D) planning system.

With 3D breast planning becoming more common, it is natural to ask whether an analogous correction scheme can be devised that uses the 3D breast shape, but remains both simple and reliable. Here, we describe such an algorithm and show that it performed well when applied to tangential breast fields from 20 patients receiving whole‐breast irradiation.

## II. METHODS

### A. Data

**Figure 1:**
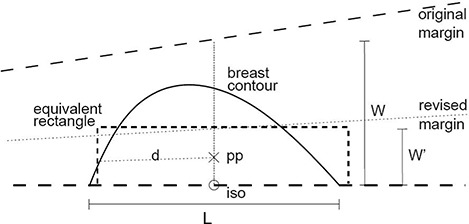
A typical breast contour (solid line) can be approximated as a rectangular solid (short‐dashed line). The width W′ is chosen so that the volume of the rectangular solid equals the irradiated volume. A half‐blocked tangential field is indicated incident from the left side. pp=prescription point; d=depth to prescription point pp; iso=isocenter.

Twenty computed tomography datasets and whole‐breast irradiation plans using tangent half‐blocked beam pairs were available for the study. Twelve plans used 6‐MV beams exclusively. Eight plans used mixed 6‐MV and 15‐MV beams, with the same geometry for both energies on each of the lateral and medial beams. Dose was prescribed to a point approximately midway between the beam entry points and one third of the distance from the posterior field border to the anterior breast surface (Fig. 1). Plans were calculated using the ${\text{Pinnacle}}^{3}$ (Philips Medical Systems, Andover, MA) planning system's collapsed cone algorithm with heterogeneity corrections to account for the low density of lung tissue. Plans were exported to RadCalc (Lifeline Software, Tyler, TX) for a second check of monitor units.

For each beam, the entry–exit distance along the central axis was recorded (*L* in Fig. 1), as was the equivalent depth to the prescription point $\left(d_{\text{eff}}\right)$, the jaw settings (*W, H*), and the required monitor units as calculated by both ${\text{Pinnacle}}^{3}$ and RadCalc.

The proposed correction factor requires an estimate of the volume of tissue irradiated by each beam. The external body contour was constructed using the automated contouring tools in ${\text{Pinnacle}}^{3}$, and beam margins were contoured manually. A composite structure was defined from the intersection of those two structures. The volume of this composite structure is an estimate of the irradiated volume, consisting of breast tissue plus small amounts of lung and chest wall. We also estimated the irradiated volume by autocontouring isodose surfaces. These estimates of the irradiated volume were also recorded for each field.

### B. Defining a correction factor

In a hand calculation assuming full scatter conditions, the dose *D* to the prescription point PP is estimated^(5)^ as

(1) $$D=k\cdot \text{MU}\cdot S_{c}(F)\cdot S_{p}(F)\cdot \text{TMR}(d_{\text{eff}},F)\cdot \text{OAF}(d_{\text{eff}},\rho )\cdot \text{WF}\cdot {\left(r_{0}/r_{\text{pp}}\right)}^{2},$$

where *k* is the dose per monitor unit at the calibration depth; $S_{c}$ is the collimator scatter factor; $S_{p}$ is the phantom scatter factor; TMR is the tissue‐maximum factor; OAF is an off‐axis correction; WF is the wedge factor; and ${\left(r_{0}/r_{\text{pp}}\right)}^{2}$ is the inverse square correction. The $d_{\text{eff}}$ is the radiologic or effective depth of the point PP, and ρ is the off‐axis distance, with $r_{0}$ being the calibration point distance, and $r_{\text{pp}}$ the distance from the source to the point PP. *F* is the equivalent square field dimension related to a rectangular field W×H by the familiar relation

(2) F=2WH/(W+H).

Because of the missing tissue in breast tangent fields, equation 1 overestimates the dose for a given number of monitor units. However, for some W′ smaller than *W*, scatter dose to point PP would be smaller, and equation 1 would predict the “correct” dose. In particular, we wish to estimate W′ assuming that full scatter conditions are present.

Consider reducing *W* to W′ as indicated in Fig. 1 and repeating the calculation. This new field over depth *L* contains an irradiated volume V′ defined by

- a plane containing the central axis,
- three divergent field margins defining a field W′×H at the isocenter distance *f*, and
- two planes perpendicular to the central axis at distances f−L/2 and f+L/2.

This half of a pyramidal frustum contains a volume V′ ^(6)^:

(3) $$V\prime =HW\prime L\;(1+(1/3)\;{\left(L/2f\right)}^{2}).$$

In our data set, the average value of *L* is 22.5 cm, and *f* is 100 cm, so that

(4) V′≈W′HL,

to better than 0.4%, which is the volume of a rectangular prism with depth *L* and cross section equal to the field dimensions *H* and W′ at isocenter. If we choose W′ such that V′=V, then

(5) W′=V/LH,

and this new field has an equivalent square dimension

(6) F′=2HW′ /(H+W′).

Calculation of the dose to point PP can then be revised to reflect the smaller volume of phantom irradiated by replacing *F* with F′ in the terms $S_{p}$ and TMR. None of the other terms in equation 1 change; in particular, $S_{c}$ is unchanged, because the physical jaws are not moved. To deliver the same dose *D* to point PP requires ${\text{MU}}^{\prime }$ monitor units, as defined by

(7) $$D=k\cdot \text{MU}\prime \cdot S_{c}(F)\cdot S_{p}(F\prime )\cdot \text{TMR}(d_{\text{eff}},F\prime )\cdot \text{OAF}(d_{\text{eff}},\rho )\cdot WF\cdot {\left(r_{0}/r_{\text{pp}}\right)}^{2}.$$

Comparing equations 1 and 7, a correction factor

(8) $$f_{\text{cor}}=\text{MU}\prime \;/\;\text{MU}=[S_{p}(F)\cdot \text{TMR}(d_{\text{eff}},F)]/[S_{p}(F\prime )\cdot \text{TMR}(d_{\text{eff}},F\prime )]$$

can be defined analogous to that defined previously.^(^ ^2^ ^,^ ^3^ ^)^ We applied this multiplicative factor $f_{\text{cor}}$ to the MU estimate from RadCalc and compared the result to the ${\text{Pinnacle}}^{3}$ monitor units.

## III. RESULTS AND DISCUSSION

Table 1 summarizes the average, standard deviation, and maximum and minimum differences between the ${\text{Pinnacle}}^{3}$ and RadCalc monitor units before and after application of $f_{\text{cor}}$ (equation 8). The corrected ${\text{MU}}^{\prime }$ was not rounded before the percentage difference was calculated. The mean difference in MUs for all beams was reduced from 5.0% to −0.5%, and the standard deviation remained essentially unchanged.

**Table 1:** Summary of differences in monitor units (MUs) between ${\text{Pinnacle}}^{3}$ (Philips Medical Systems, Andover, MA) and RadCalc (Lifeline Software, Tyler, TX) before and after application of the proposed correction factor^a^

	Difference in MUs at various energies					
	Before correction			After correction		
	All	6 MV	15 MV	All	6 MV	15 MV
Average (%)	5.0	5.5	3.7	−0.5	−0.7	0.1
Standard deviation (%)	1.2	0.8	1.0	1.1	1.2	0.9
Maximum (%)	7.1	7.1	6.1	2.0	1.5	2.0
Minimum (%)	2.1	3.3	2.1	−3.7	−3.7	−1.2
Beams (*n*)	56	40	16	56	40	16

a Monitor units have not been rounded to an integer after application of the correction.

**Figure 2:**
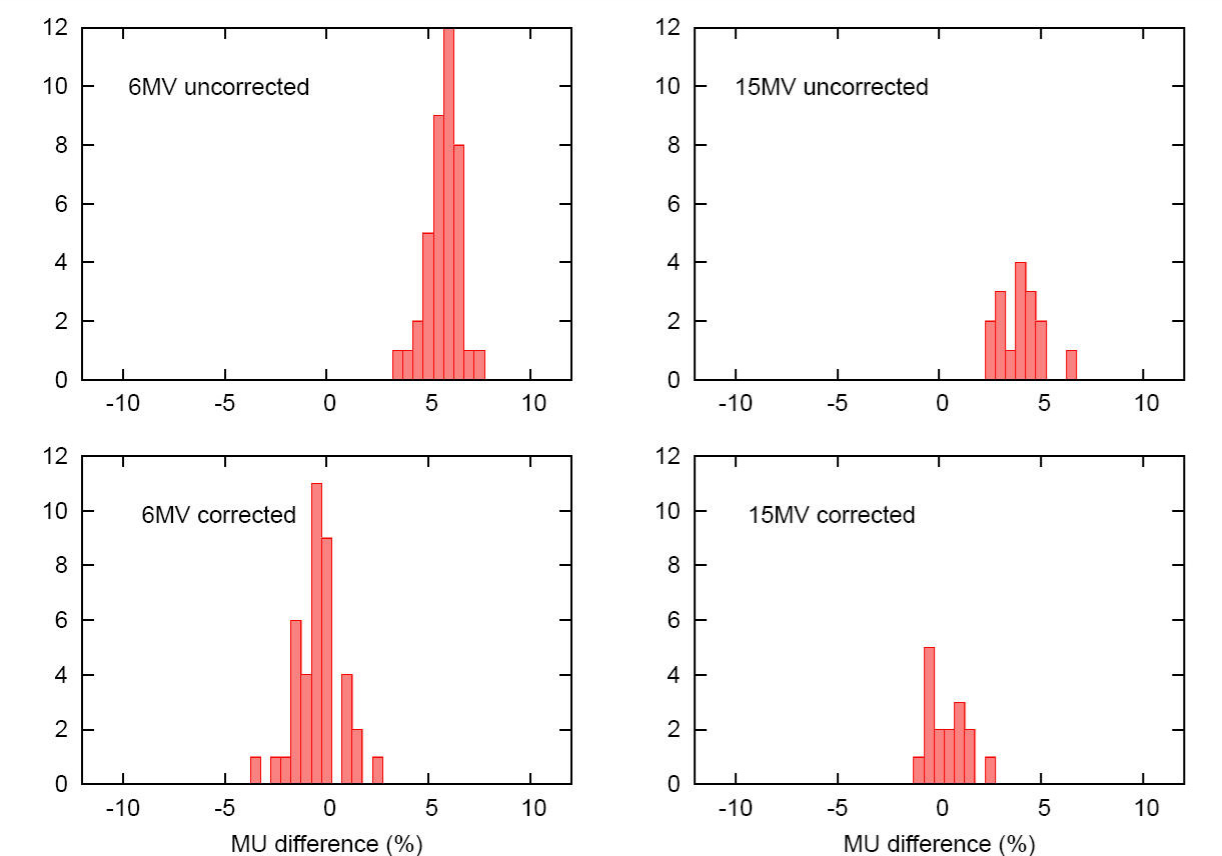
The histograms in the upper row represent the differences between monitor units (MUs) predicted by a three‐dimensional planning system and a MU‐checking software or hand calculations, assuming full scatter conditions at two energies. The histograms in the lower row show the distribution of discrepancies in MUs after the proposed correction factor has been applied.

**Figure 3:**
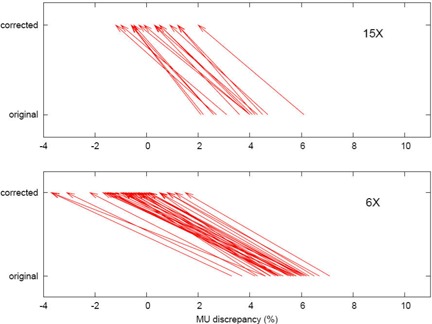
For each energy, two horizontal 1−d scatter plots are shown, the lower for the uncorrected monitor unit (MU) check, and the upper for the corrected MU check. The vectors indicate the correction for MUs for each field.

The correction factor is energy‐dependent: the average deviation at 6 MV is reduced to −0.7% from 5.5%, and at 15 MV, to 0.1% from 3.7%. The correction factor slightly increased the standard deviation of the 6‐MV discrepancies and left the 15‐MV standard deviation essentially unchanged. Fig. 2 shows histograms of the monitor unit discrepancy, by energy, before and after correction. Fig. 3 provides a more dramatic representation of the correction, with the 1−d scatter plots of the deviations being linked by vectors showing the individual corrections for each field.

The correction method presented here successfully corrects for the missing scatter dose to the prescription point. However, replacing the irregularly shaped breast with a rectangular prism of equivalent volume is not strictly correct, because the scatter is not isotropic. That approach is analogous to replacing an irregular field with a square field of equal area rather than with “an equivalent square.”

In the case of breast tangent fields, this “rectangular” approximation fills in some of the upstream missing tissue, increasing scatter toward the calculation point and thus decreasing the monitor unit estimate. RadCalc or hand calculations also do not make any allowance for lack of scatter from the lung, which would further reduce the monitor unit estimate. However, the use of the smaller effective or radiologic depth has the effect of shifting the rectangular prism of material downstream, as we have tried to show in Fig. 1. That approach works to increase the prediction of required monitor units. It appears that, to first order, these effects combine to produce a very successful correction strategy. There remains a smaller but significant variation in the slopes of the vectors (Fig. 3). That effect must result in part from variations in the choice of prescription point in each plan, in part from variations in the breast contours, and in part from an oversimplified treatment of the physics. The correction should therefore not be relied on for primary calculation of monitor units, but only for a scatter correction to second checks.

Determination of the irradiated volume requires two extra steps in the planning process: contouring of the body and definition of the beam margins. The body contour is easily constructed based on a threshold value. Many treatment planning systems include such an automated tool, and the extra cost in time is minimal. Contouring of the beam outline requires manual contours on a few slices at the superior and inferior divergent margins, and the planning software is able to interpolate in the midrange. Total time for this contouring and combining to produce an irradiated volume was less than 5 minutes for two fields.

A simpler method of estimating irradiated volume is to contour an isodose level. We found that the volume enclosed by the 30% isodose surface correlated well with the volume irradiated by each beam ($r^{2}=0.979$ on 40 points), with a mean difference of 4%. Estimates of *W*'—and hence $f_{\text{cor}}$—were essentially unchanged. The 30% of prescription volume was chosen somewhat arbitrarily. The volume is produced by the sum of two beams and not by the individual irradiated volume for each beam, but the average 4% difference is not significant. Consider that the average revised field width *W*' was 4.5 cm; the average field length, 23 cm; and the average effective depth, 8 cm. A 4% change in *W*' then corresponded to a change in *F*' to 7.3 cm from 7.5 cm on average. At 6 MV, that change alters $S_{p}$ by ~0.1% and TPR by ~0.3%. Thus isovolume contouring is sufficiently accurate for estimating *W*' as well.

Table 2 compares our proposed correction with previous suggestions by Kay and Dunscombe^(3)^ and by Prado et al.^(4)^ The previously suggested methods use only one contour and do not incorporate curvature in the orthogonal direction, underestimating the amount of missing tissue. Our proposed correction factor is larger, and the unresolved difference in monitor units after correction is smaller, suggesting a more accurate account of the deviation from full scatter conditions.

**Table 2:** Comparison of the proposed correction factor with those previously described by Kay and Dunscombe^(3)^ and Prado et al.^(4)^

	Proposed correction		Kay and Dunscombe correction		Prado et al. correction	
	6 MV	15 MV	6 MV	15 MV	6 MV	15 MV
Correction factor^a^	1.065±0.008	1.037±0.005	1.033±0.004	1.015±0.003	1.038±0.005	1.019±0.002
Remaining difference in MUs after correction ^b^ (%)	−0.7±1.2	0.1±0.9	2.3±0.9	2.2±1.0	1.8±1.0	1.9±1.0

a The average and standard deviation (1σ) of the calculated correction factors.

b The remaining unresolved difference in monitor units (MUs), tabulated for the forty 6‐MV and sixteen 15‐MV fields available in the present study.

## IV. CONCLUSIONS

Previous strategies^(^ ^3^ ^,^ ^4^ ^)^ for reconciling monitor unit calculations for 2.5D planning of tangent breast fields have been shown to extend to 3D whole‐breast plans, reducing the residual disagreements to less than 1%. The simplified correction presented here appears to work well for the geometry encountered in breast treatments, and the additional work required of the planner is limited to obtaining the chord length along the central axis and an estimate of the irradiated volume. A clinical physicist may choose to implement this correction method or to use it as the basis for justifying a modification of the plan acceptance criteria after validation for local procedure and treatment planning systems.

## Supporting information

Supplementary Material

## References

[1] Kutcher GJ , Coia L , Gillin M , et al. Comprehensive QA for radiation oncology: report of AAPM Radiation Therapy Committee Task Group 40. Med Phys. 1994; 21 (4): 581–618. 8058027 10.1118/1.597316

[2] Ayyangar KM , Saw CB , Gearheart D , Shen B , Thompson R . Independent calculations to validate monitor units from ADAC treatment planning system. Med Dosim. 2003; 28 (2): 79–83. 12804704 10.1016/S0958-3947(02)00237-6

[3] Kay I , Dunscombe P . Verifying monitor unit calculations for tangential breast fields. J Appl Clin Med Phys. 2006; 7 (4): 50–57. 10.1120/jacmp.v7i2.2177 PMC5722436 17533323

[4] Prado KL , Kirsner SM , Erice RC . Corrections to traditional methods of verifying tangential‐breast 3D monitor‐unit calculations: use of an equivalent triangle to estimate effective fields. J Appl Clin Med Phys. 2003; 4 (1): 51–57. 12540818 10.1120/jacmp.v4i1.2541 PMC5724436

[5] Khan FM . The physics of radiation therapy. 3rd edition Philadelphia (PA): Lippincott Williams and Wilkins; 2003 560 p.

[6] Beyer WH, editor. CRC standard mathematical tables. 25th edition Boca Raton (FL): CRC Press; 1978: 146.

