# Supplementary material for: Verifying monitor unit calculations for tangential whole‐breast fields in three‐dimensional planning
Source: J Appl Clin Med Phys. 2008 Jan 28;9(1):47–53. doi: 10.1120/jacmp.v9i1.2713 (PMC5721530; doi:10.1120/jacmp.v9i1.2713)
Supplement: Supplementary file 1 — Supplementary Material [file ACM2-9-47-s001.doc]

**Verifying Monitor Unit Calculations for Tangential Whole Breast Fields in 3D Planning.**

**Abstract:** An essential component of quality assurance in radiation therapy is verifying the accuracy of monitor unit calculations. For tangential breast fields, monitor unit differences between primary calculations and second checks are usually larger than considered acceptable at other anatomical sites. A simple model to reconcile the differences between sophisticated and simple algorithms is presented, based on estimating the volume irradiated by the field, replacing the breast contour with a rectangular block having an equal volume but using a new field width which provides almost equivalent scatter to the prescription point. This analysis can also assist the treatment planning physicist in selecting a tolerance window for verifying monitor unit calculations for tangential breast fields.

Key Words: radiotherapy, breast, tangent field, treatment planning, quality assurance, monitor-unit calculation.

----------------------------

PACS numbers: 87.53.Kn, 87.53.Tf, 87.53.Xd

**I Introduction**

External beam therapy quality assurance (QA) requires that plans be validated^1^, and an independent monitor unit calculation is an essential part of that QA process. Tangent fields used for breast treatment always present a challenge to the physicist performing plan checks, particularly verifying the monitor units. Breast tangent fields combine complex external contours and field borders outside the body, resulting in a significant amount of “missing tissue”. Monitor unit verification is usually performed with simple algorithms that assume full scatter conditions. The result is an overestimate of scatter dose to the calculation point in the second check and an underestimate of the monitor units required to deliver the desired dose.

Various algorithms (eg., Ayyangar et al.^1^) have been devised to estimate and remove this discrepancy. Kay and Dunscombe^3^ presented a simplified method for 2.5 dimensional (2.5D) breast tangent fields. This method estimated the cross-sectional area of irradiated breast and determined the field dimensions which would irradiate a rectangle of equal area. A correction factor was calculated based on a ratio of phantom scatter factors (S_p_) and the tissue maximum ratios (TMR) using the original and revised field widths. Accuracy of this correction factor was similar to that of Ayyangar et al.^1^, and was simpler to calculate. Prado et al.^4^ proposed a similar technique using a triangular approximation of the superior-inferior external breast contour as seen in a beam's-eye-view. Their method corrected monitor unit calculations based on full scatter conditions to within 2% of those calculated using a 3D planning system.

With 3D breast planning becoming more common, it is natural to ask whether an analogous correction scheme can be devised which uses the 3D breast shape but remains both simple and reliable. In this note we describe such an algorithm and show that it performed well when applied to tangential breast fields from 20 patients receiving whole breast irradiation.

**II Methods**

*Data:*

Twenty CT datasets and whole breast irradiation plans using tangent half blocked beam pairs were available for this study. Twelve plans used 6MV beams exclusively. Eight plans used mixed 6 and 15MV beams, with the same geometry for both energies on each of the lateral and medial beams. Dose was prescribed to a point approximately midway between the beam entry points and 1/3 of the distance from the posterior field border to the anterior breast surface (Fig. 1). Plans were calculated using the Pinnacle^3^ (Philips Medical Systems) planning system's collapsed cone algorithm with heterogeneity corrections to account for the low density of the lung tissue. Plans were exported to RadCalc (Lifeline Software Inc.) for second checking of monitor units.

For each beam the entry/exit distance along the central axis was recorded (L in Fig. 1), as was the equivalent depth to the prescription point (d_eff_), the jaw settings (W and H) and the required monitor units as calculated by both Pinnacle^3^ and RadCalc.

The proposed correction factor requires an estimate of the volume of tissue irradiated by each beam. The external body contour was constructed using the automated contouring tools in Pinnacle^3^ and beam margins were contoured manually. A composite structure was defined from the intersection of these two structures. The volume of this composite structure is an estimate of the irradiated volume consisting of breast tissue as well as small amounts of lung and chest wall. We also estimated the irradiated volume by autocontouring isodose surfaces. These estimates of the irradiated volume were also recorded for each field.

*Defining a correction factor:*

In a hand calculation assuming full scatter conditions, the dose D to the prescription point PP is estimated as^5^

D = k ⋅ MU ⋅ S_c_(F) ⋅ S_p_(F) ⋅ TMR(d_eff_, F) ⋅ OAF(d_eff_, ρ) ⋅ WF ⋅ (r_0_/r_pp_)^2^, (1)

where k is the dose per monitor unit at the calibration depth, S_c_ the collimator scatter factor, S_p_ the phantom scatter factor, TMR the tissue-maximum factor, OAF an off-axis correction, WF the wedge factor and the last term an inverse square correction. d_eff_  is the radiological or effective depth of the point PP, r is the off-axis distance, r_0_ the calibration point distance and r_pp_ the distance from the source to the point PP. F is the equivalent square field dimension related to a rectangular field W x H by the familiar relation

F = 2WH/(W+H). (2)

Since there is missing tissue in the breast tangent fields, equation (1) overestimates the dose for a given number of monitor units. However for some W' smaller than W, scatter dose to the point PP would be smaller, and equation (1) would predict the 'correct' dose. In particular, we wish to estimate W' assuming full scatter conditions are present.

Consider reducing W to W' as indicated in Fig. 1 and repeating the calculation. This new field over depth L contains an irradiated volume V' defined by: a plane containing the central axis, three divergent field margins defining a field W' by H at the isocentre distance f and two planes perpendicular to the central axis at distances f-L/2 and f+L/2. This half a pyramidal frustrum contains a volume^6^

V' = HW'L(1+(1/3)(L/2f)^2^) . (3)

In our data set the average value of L is 22.5 cm, and f = 100 cm, so

V' ≈ W' H L (4)

to better than 0.4%, which is the volume of a rectangular prism depth L and cross section equal to the field dimensions H and W' at isocentre. If we choose W' such that V' = V, then

W' = V/LH, (5)

and this new field has an equivalent square dimension

F' = 2 H W' / (H + W'). (6)

Calculation of the dose to point PP can then be revised to reflect the smaller volume of phantom irradiated by replacing F by F' in the terms S_p_ and TMR. None of the other terms in equation (1) change; in particular S_c_ is unchanged as the physical jaws are not moved. To deliver the same dose D to point PP requires MU' monitor units as defined by:

D = k ⋅ MU' ⋅ S_c_(F) ⋅ S_p_(F') ⋅ TMR(d_eff_, F') ⋅ OAF(d_eff_, ρ) ⋅ WF ⋅ (r_0_/r_pp_)^2^. (7)

Comparing equations (1) and (7), one can define a correction factor

f_cor_ = MU'/MU = S_p_(F) TMR(d_eff_ , F) / S_p_(F')TMR(,d_eff_,F') (8)

analogous to that defined previously^2,3^. This multiplicative factor f_cor_ was applied to the MU estimate from RadCalc and compared to the Pinnacle^3^ monitor units.

**III. Results & Discussion**

Average, standard deviation, maximum and minimum differences between Pinnacle^3^ and RadCalc monitor units are summarized inTable 1, before and after application of f_cor_ (equation 8). The corrected MU' was not rounded before the percent difference was calculated. The mean difference in MUs for all beams was reduced from 5.0% to -0.5% and the standard deviation remained essentially unchanged.

The correction factor is energy dependent; the average deviation at 6MV is reduced from 5.5% to -0.7%, and at 15MV from 3.7% to 0.1%. The correction factor slightly increased the standard deviation of the 6MV discrepancies, and left the 15MV standard deviation essentially unchanged. Histograms of the monitor unit discrepancy, by energy, before and after correction are shown in Fig. 2. A more dramatic representation of the correction is provided in Fig. 3, where the 1-d scatter plots of the deviations are linked by vectors showing the individual corrections for each field.

The correction method presented successfully corrects for the missing scatter dose to the prescription point. However, replacing the irregularly shaped breast with a rectangular prism of equivalent volume is not strictly correct, since the scatter is not isotropic. This would be analogous to replacing an irregular field with a square field of equal area rather than using the 'equivalent square'.

In the case of breast tangent fields, this 'rectangular' approximation fills in some of the upstream missing tissue, increasing scatter toward the calculation point, and thus decreasing the monitor unit estimate. RadCalc or hand calculations also do not make any allowance for the lack of scatter from the lung, and this would further decrease the monitor unit estimate. However, the use of the smaller effective or radiological depth has the effect of shifting the rectangular prism of material downstream as we have tried to show in Fig. 1. This works to increase the prediction of required monitor units. It appears that to first order these effects combine to produce a very successful correction strategy. There remains a smaller but significant variation in slopes of the vectors in Fig. 3. This must be due in part to variations in the plans' choices of prescription point, in part to variations in the breast contours, and in part to an over-simplified treatment of the physics. This correction should not be relied upon for primary calculation of monitor units, but only for a scatter correction to second checks.

Determination of the irradiated volume required two extra steps in the planning process, contouring of the body and defining the beam margins. The body contour was easily constructed based on a threshold value; many treatment planning systems include such an automated tool and the extra cost in time is minimal. Contouring of the beam outline required manual contours on a few slices at the superior and inferior divergent margins, and the planning software was able to interpolate in the midrange. Total time for this contouring and combining to produce an irradiated volume was less than 5 minutes for two fields.

A simpler method of estimating irradiated volume is to contour an isodose level. It was found that the volume enclosed by the 30% isodose surface correlated well with the volume irradiated by each beam, (r^2^ = 0.979 on 40 points) with a mean difference of 4%. Estimates of W' and hence f_cor_ were essentially unchanged. The 30% of prescription volume was chosen somewhat arbitrarily. This volume is produced by the sum of two beams and not the individual irradiated volume for each beam, but the average 4% difference is not significant. Consider that the average revised field width W' was 4.5cm, the average field length was 23cm and the average effective depth was 8cm. A 4% change in W' then corresponded to a change in F' on average from 7.5 to 7.3 cm. At 6MV, this changes Sp by ~0.1% and TPR by ~0.3%. Thus iso-volume contouring is sufficiently accurate for estimating W' as well.

This proposed correction is compared with previous suggestions of Kay et al.^3^ and Prado et al.^4^ in table 2. The previous methods use only one contour and do not incorporate curvature in the orthogonal direction, underestimating the amount of missing tissue. Our proposed correction factor is larger and the unresolved difference in monitor units after correction is smaller, suggesting a more accurate account of the deviation from full scatter conditions.

**IV. Conclusions**

Previous strategies ^3,4^ for reconciling monitor unit calculations for 2.5D planning of tangent breast fields have been shown to extend to 3D whole breast plans and reduce the residual disagreements to less than 1%. This simplified correction appears to work well for the geometry encountered in breast treatments and the additional work required of the planner is limited to obtaining the chord length along the central axis and an estimate of the irradiated volume. A clinical physicist may choose to implement this correction method, or use it as the basis for justifying a modification of their plan acceptance criteria after validation for local procedure and treatment planning systems.

**References**

^1^ Kutcher GJ, Coia L, Gillin, M., et al. Comprehensive QA for radiation oncology: Report of AAPM Radiation Therapy Committee Task Group 40. Med Phys. 1994; 21(4):581-618.

^2^ Ayyangar KM, Saw CB, Gearheart D, et al. Independent calculations to validate monitor units from ADAC treatment planning system. Med. Dosim. 2003; 28(2):79-83.

^3^ Kay I, Dunscombe P. Verifying Monitor Unit Calculations for Tangential Breast Fields J Appl Clin Med Phys. 2006; 7(2):50-57.

^4^ Prado KL, Kirsner SM, Erice RC. Corrections to traditional methods of verifying tangential-breast 3D monitor-unit calculations: Use of an equivalent triangle to estimate effective fields. J Appl Clin Med Phys. 2003; 4(1):51-57.

^5^ Khan FM. The physics of radiation therapy. 3rd ed. Philadelphia (PA): Lippincott Williams and Wilkins; 2003. 560 p.

^6^ Beyer WH, editor. CRC Standard mathematical tables, 25^th^ ed. Boca Raton (FL): CRC Press, 1978: 146.

**Figure Captions**

(fig1.eps)

Fig. 1. A typical breast contour (solid) can be approximated as a rectangular solid (dashes). The width W’ is chosen so that the volume of the rectangular solid equals the irradiated volume. A half blocked tangential field is indicated incident from the left side, pp = prescription point, iso = isocentre.

(histogram.eps)

Fig. 2. The upper row of histograms represent the differences between monitor units predicted by a 3D planning system and a monitor unit checking software or hand calculations assuming full scatter conditions at two energies. The lower histograms show the distribution of discrepancies in monitor units after the proposed correction factor has been applied.

(vectorplot.pdf)

Fig. 3. For each energy, two horizontal 1-d scatter plots are shown, the lower for the uncorrected MU check, and the upper for the corrected MU check. The vectors indicate the correction for each field's MUs.

Tables:

| Difference in MUs | Before correction | | | After Correction | | |
| --- | --- | --- | --- | --- | --- | --- |
| Energy: | All | 6MV | 15MV | All | 6MV | 15MV |
| Average: | 5.0% | 5.5% | 3.7% | -0.5% | -0.7% | 0.1% |
| Standard Deviation: | 1.2% | 0.8% | 1.0% | 1.1% | 1.2% | 0.9% |
| Max: | 7.1% | 7.1% | 6.1% | 2.0% | 1.5% | 2.0% |
| Min: | 2.1% | 3.3% | 2.1% | -3.7% | -3.7% | -1.2% |
| Number of beams: | 56 | 40 | 16 | 56 | 40 | 16 |

Table 1: Summary of differences in monitor units between Pinnacle^3^ and RadCalc before and after the proposed correction factor is applied. (Monitor units have not been rounded to an integer after applying the correction.)

|  | Proposed Correction | | Correction of Kay et al.^3^ | | Correction of Prado et al.^4^ | |
| --- | --- | --- | --- | --- | --- | --- |
| Energy | 6MV | 15MV | 6MV | 15MV | 6MV | 15MV |
| correction factor | 1.065±0.008 | 1.037±0.005 | 1.033±0.004 | 1.015±0.003 | 1.038±0.005 | 1.019±0.002 |
| remaining difference in MUs after correction | -0.7±1.2% | 0.1±0.9% | 2.3±0.9% | 2.2±1.0% | 1.8±1.0% | 1.9±1.0% |

Table 2: Comparison of the proposed correction factor compared with those previously described in Kay et al.^2^ and Prado et al.? The average and standard deviation (1σ) of the calculated correction factors and the remaining unresolved difference in MUs is tabulated for the forty 6MV and sixteen 15MV fields available in this study.
